# Consolidation/reconsolidation therapies for the prevention and treatment of PTSD and re-experiencing: a systematic review and meta-analysis

**DOI:** 10.1038/s41398-021-01570-w

**Published:** 2021-09-03

**Authors:** Laurence Astill Wright, Louise Horstmann, Emily A. Holmes, Jonathan I. Bisson

**Affiliations:** 1 Division of Psychological Medicine and Clinical Neurosciences, Cardiff University School of Medicine, Cardiff, UK; 2 Department of Psychology, Uppsala University, Uppsala, Sweden

**Keywords:** Pathogenesis, Psychiatric disorders

## Abstract

Translational research highlights the potential of novel 'memory consolidation/reconsolidation therapies' to treat re-experiencing symptoms and post-traumatic stress disorder (PTSD). This systematic review and meta-analysis assessed the efficacy of so-called memory consolidation/reconsolidation therapies in randomised controlled trials (RCTs) for prevention and treatment of PTSD and symptoms of re-experiencing in children and adults (PROSPERO: CRD42020171167). RCTs were identified and rated for risk of bias. Available data was pooled to calculate risk ratios (RR) for PTSD prevalence and standardised mean differences (SMD) for PTSD/re-experiencing severity. Twenty-five RCTs met inclusion criteria (16 prevention and nine treatment trials). The methodology of most studies had a significant risk of bias. We found a large effect of reconsolidation interventions in the *treatment* of PTSD (11 studies, *n* = 372, SMD: −1.42 (−2.25 to −0.58), and a smaller positive effect of consolidation interventions in the prevention of PTSD (12 studies, *n* = 2821, RR: 0.67 (0.50 to 0.90). Only three protocols (hydrocortisone for PTSD prevention, Reconsolidation of Traumatic Memories (RTM) for treatment of PTSD symptoms and cognitive task memory interference procedure with memory reactivation (MR) for intrusive memories) were superior to control. There is some emerging evidence of consolidation and reconsolidation therapies in the prevention and treatment of PTSD and intrusive memories specifically. Translational research should strictly adhere to protocols/procedures describing precise reconsolidation conditions (e.g. MR) to both increase the likelihood of positive findings and more confidently interpret negative findings of putative reconsolidation agents.

## Introduction

Scientific advances over the previous 20 years have demonstrated that, contrary to previous thinking, memories are highly malleable and that this may have applications for therapeutic innovation [1, 2]. Basic scientific research into the neurobiology of memory formation (initial memory consolidation) and memory reconsolidation (for older memories) suggests a hypothetical process whereby memory is retrieved and altered [3, 4]. Translational clinical studies have aimed to utilise such basic scientific advances in memory modification mechanisms (consolidation and reconsolidation) to prevent and treat psychopathology—particularly the re-experiencing of distressing traumatic memories and post-traumatic stress disorder (PTSD) [1, 5–8]. In 2000, Nader et al. [3] ‘s seminal paper demonstrated that neuroplasticity and protein synthesis are not only required for the consolidation of new emotional memories but also when already consolidated fear memories are retrieved. The process of reconsolidation can lead to three outcomes: no change, a stronger memory or a weaker memory. That is, the hypothesis suggests that once retrieved (e.g. via memory reactivation (MR)), new protein synthesis can alter old memories, and reform them without their previous emotional salience via reconsolidation update mechanisms. While Misanin et al. [9] showed post-retrieval memory modification was possible with electroconvulsive shocks, Nader et al [3]. demonstrated that the protein synthesis antagonist anisomycin can be given to block memory reconsolidation and produce amnesia in rodents. Anisomycin is toxic in humans, but the work provides insights into the mobilisation and adaptation of emotional memories as a treatment after trauma [10, 11].

Such insights in memory modification/cognitive science [12] have led to various novel therapies emerging, that here we term 'memory consolidation/reconsolidation therapies'—therapies where the development or delivery of the therapeutic modality has been informed by reconsolidation theory and with the stated aim of targeting reconsolidation/consolidation. So-called consolidation/reconsolidation therapies include heterogeneous methods such as pharmacological interference with the reconsolidation of the destabilised memory. Here we group these into three broad domains—(i) those using pharmacological agents (e.g. propranolol [13], hydrocortisone [14]), (ii) those using psychological therapy techniques e.g. The Rewind Technique [15], Reconsolidation of Traumatic Memories (RTM) [16] and (iii) behavioural consolidation studies using cognitive tasks e.g. cognitive task memory interference procedure with MR [17].

Some consolidation/reconsolidation therapies have primarily targeted intrusive images and re-experiencing phenomena [16, 17]. This focused symptom approach might be useful in its own right (given there are 636,120 different ways to have PTSD [18]). Reducing intrusive symptoms may also have useful downstream effects since they are hypothesised to be a mediator of other PTSD symptom clusters [19, 20]). A second reason to take a single symptom approach is that it can represent a more direct translation of developments in experimental/cognitive science [1]. New targets and stepwise adaptation from lab procedures may be useful as to date many approaches to prevent and treat the full syndrome of PTSD have failed or are inaccessible [21, 22]. Here we adopt the terminology of consolidation and reconsolidation therapies to describe specific novel interventions, though some established PTSD therapies may work through similar mechanisms. For example, it has long been argued that trauma-focused cognitive behavioural therapy (TF-CBT) [23] and eye movement desensitisation and reprocessing (EMDR) [23] may ameliorate PTSD symptoms partly via extinction, but they also aim to modify an old trauma memory following reactivation, thus, arguably, could be working at least in part via reconsolidation update mechanisms. On the other hand, some of the studies we consider in this review are included as they purport to use 'reconsolidation' by some clinicians and researchers even though may not fully meet some fundamental requirements of reconsolidation theory e.g. RTM, which involves rewinding techniques [16]. To summarise, this review has included studies where the development or delivery of a therapeutic modality has been informed by reconsolidation theory, regardless of the modality of the intervention (e.g. pharmacological/psychological therapy/task-based) and with the stated aim of targeting reconsolidation/consolidation. This systematic review aimed to summarise the evidence for and assess the efficacy of so-called consolidation/reconsolidation therapy RCTs in the prevention and treatment of PTSD, PTSD symptoms and intrusive symptoms.

## Methods

We adopted a methodology based on the Cochrane Handbook for Systematic Reviews of Interventions [24] and completed a PRISMA checklist (Supplementary Table 1). The study was registered and adhered with the International Prospective Register of Systematic Reviews (PROSPERO: CRD42020171167) [25].

We cannot directly measure if consolidation/reconsolidation has occurred in human participants, and here (as discussed above) we only included therapies which specifically tried to target consolidation/reconsolidation i.e. drawn on this theory as a hypothesised mechanism of change. This, however, provides no assurance that reconsolidation has been achieved. Most reconsolidation therapies and some consolidation therapies attempted to trigger reactivation of the traumatic memory, as this is a prerequisite to reconsolidation [2, 26], with the stated aim of targeting reconsolidation/consolidation, although we must remain agnostic about the true underlying mechanism of action.

Previous basic and clinical papers have not paid great attention to the role of MR within such procedures, with some experimental studies missing it out completely [27]. It is however critical from a theoretical perspective and warrants highlighting here. MR will have occurred in some consolidation studies via an external cue (e.g. a prompt in psychotherapy; or memory reminder cue before a cognitive task) and in others via a memory orientation context cue [2] (e.g. the therapy was administered in the same place as their trauma occurred. Reviewing this literature highlights that procedural details may not be clear within the paper and may be useful to find ways of recording and training procedures as apparently small differences may have large impacts.

We included both prevention and treatment of PTSD consolidation/reconsolidation therapies as they include some mechanistic similarities, with both attempting to draw on consolidation/reconsolidation theory to alter a memory within a certain timeframe. The therapies themselves have attempted to target consolidation/reconsolidation in a variety of ways.

### Selection criteria

The inclusion criteria were: (a) any RCT (including cluster and cross-over trials); (b) investigating the effects of a consolidation/reconsolidation-based prevention or treatment intervention; (c) when compared to placebo, pharmacological or psychosocial interventions; (d) in participants exposed to a traumatic event with PTSD or acute stress disorder (ASD) symptoms measured using one or more validated clinician-administered or self-report outcome measures. Both published and unpublished studies were eligible for inclusion. Only studies published in English were included.

### Search strategy and selection criteria

The complete search strategy is included in the appendix. All abstracts were appraised by two independent screeners (L.A.W. and L.H.) and any disagreements were discussed. The full text of any potentially relevant papers was acquired and if we were unable to locate the full text for any study, we contacted the corresponding author to request the paper. To determine if potentially relevant studies met the inclusion criteria the full text was independently reviewed by two authors (L.A.W. and L.H.).

### Data extraction

Data were extracted by two independent reviewers using identical data extraction forms (L.A.W. and L.H.) (first extracted 08/05/20). Any irregularities between reviewers was discussed and consensus agreed on with reference to a third reviewer if necessary (J.I.B.).

### Assessment of study bias

The Cochrane Collaboration’s tool for assessing the risk of bias in randomised trials [28] was used for each study. The risk of bias was assessed by two independent reviewers (L.A.W. and L.H.) and any disagreement was resolved by discussion. A GRADE judgement (which assesses the quality of evidence to make recommendations for clinical practice [29]) was presented for each outcome.

### Synthesis of results

The primary outcome in the meta-analysis for preventative studies was a reduction in PTSD incidence and/or severity of re-experiencing symptoms 3–6 months after the traumatic event, while for treatment studies the primary outcome was a reduction in PTSD incidence and/or severity of re-experiencing symptoms 3–6 months post-intervention (it was agreed a priori that the nearest time point to this would be accepted and the vast majority of included reconsolidation studies only reported outcomes in the 1–4 weeks post-intervention). This timeframe was chosen to demonstrate the stability of treatment effects, which unfortunately could not be assessed by some studies. For PTSD/re-experiencing severity we calculated SMD and for PTSD incidence we calculated RR, along with associated confidence intervals.

For outcomes included in more than one study, we measured statistical heterogeneity by calculating the I2 statistic. An I2 of less than 30% was taken to indicate mild heterogeneity and a fixed-effects model was used. When the I2 was greater or equal to 30% a random-effects model was used. Data were collated from different consolidation/reconsolidation therapies separately according to their modality, as well as pooling the data. All analyses were performed using the Cochrane Collaboration’s Review Manager 5.3 software [30].

## Results

**Fig. 1:**
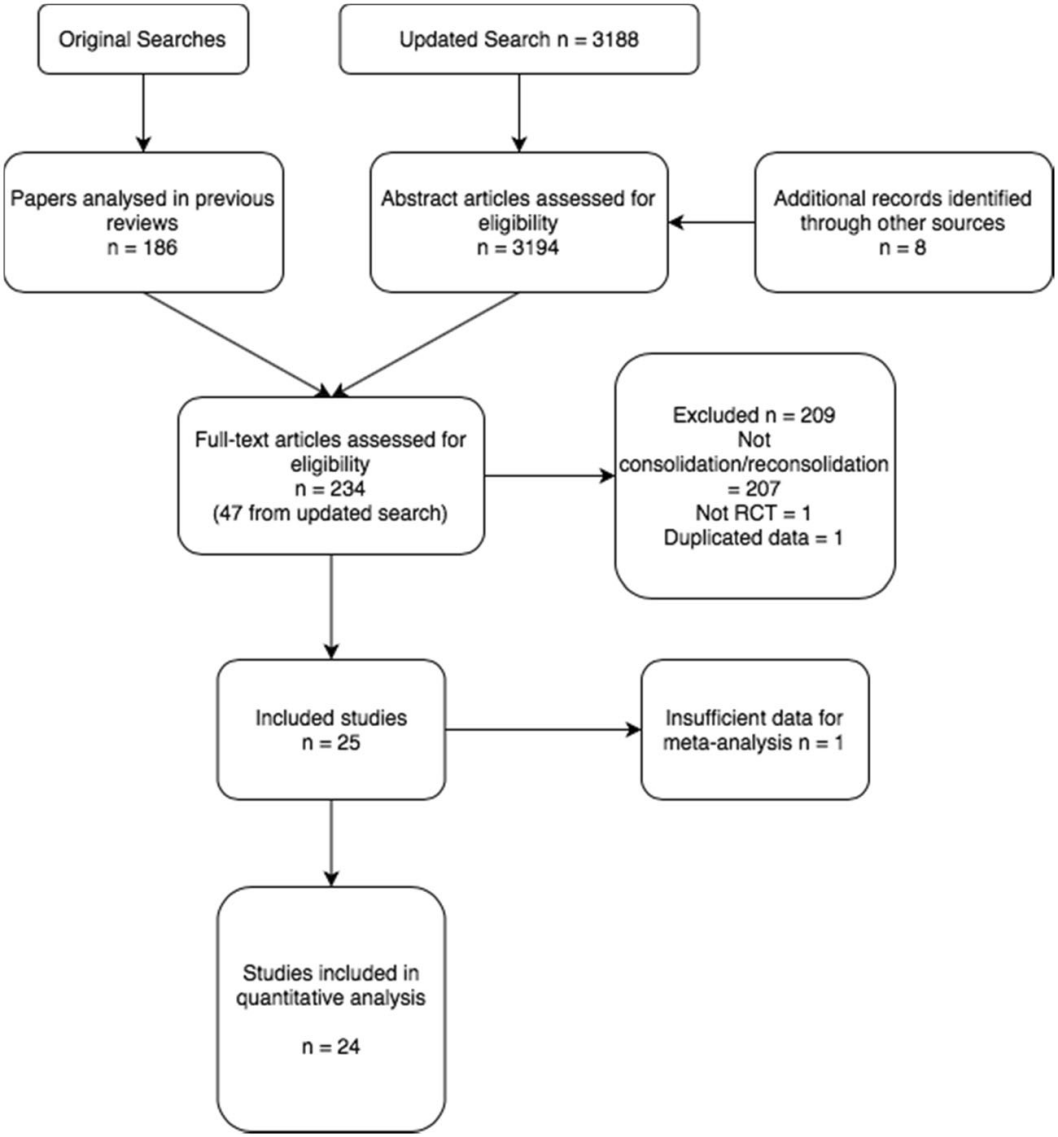
PRISMA flow diagram.

Previous searches [21–23, 31–33] produced 186 papers for analysis. The updated search contained 3188 papers. We examined the full text of 48 papers and 25 of these met the inclusion criteria. The other 23 were excluded as per Fig. 1.

Thirteen of the included studies were identified in the original searches [34–46], four from the updated search [13, 16, 17, 47], five from reference checking [10, 14, 48–50], and three provided by the authors themselves [51–53]. The 25 included studies included 2121 participants, with 16 prevention trials (*n* = 1789) and nine treatment trials (*n* = 432). One study was excluded from the meta-analysis as it lacked sufficient information [42]. Only one study assessed children/adolescents [39]. Tables 1 and 2 display detailed characteristics of the trials.

**Table 1:** Characteristics of included reconsolidation studies.

Study	Country	Trauma sample	Reconsolidation procedure	Comparator	No. of sessions	*n*	PTSD outcome	Follow up	Primary outcome
Adult pharmacological/ECT reconsolidation									
Brunet et al. 2018*	Canada	Unspecified	Propranolol & MR	Placebo & MR	6	60	PTSD severity (CAPS & PCL-C)	1 week, 6 months	1 week PTSD severity
Wood et al. 2015 Study 1^a^	USA	Combat-related	Propranolol & MR	Propranolol	1	18	PTSD severity (IES-R)	1 week	1 week PTSD severity
Wood et al. 2015 Study 2^a^	USA	Combat-related	Mifepristone & MR	Mifepristone	1	28	PTSD Severity (IES-R)	1 week	1 week PTSD Severity
Wood et al. 2015 Study 3^a^	USA	Combat-related	Mifepristone & d-cycloserine & MR	Double placebo	1	31	PTSD severity (IES-R)	2 weeks	2 week PTSD severity
Suris et al. 2010^a^	USA	Combat-related	Hydrocortisone & MR	Placebo & MR	1	19	PTSD severity (IES-R)	1 week	1 week PTSD severity
Suris et al. 2013^a^	USA	Combat-related	Sirolimus & MR	Placebo & MR	1	51	PTSD severity (CAPS & PCL)	1, 3 months	1 & 3 month PTSD severity
Corchs et al. 2018^a^	Brazil	Unspecified	ECT & MR (traumatic)	ECT & MR (neutral)	6	8	PTSD severity (DTS)	1 month	Skin conductance responses and subjective reactivity to trauma recollection
Adult psychological reconsolidation									
Gray et al. 2017^a^	USA	Mixed	RTM	Waitlist control	3	69	PTSD severity (PCL-M)	2 weeks	2 week PTSD severity
Gray et al. 2015^a^	USA	Mixed	RTM	Waitlist control	3-5	31	PTSD severity (PCL-M)	2 weeks	2 week PTSD severity
Gray et al. Unpublished^a^	USA	Mixed	RTM	Waitlist control	≤ 3	29	PTSD severity (PCL-M, PSS)	2 weeks	2 week PTSD severity
Tylee et al. 2017^a^	USA	Combat-related	RTM	Waitlist control	3	27	PTSD severity (PCL-M, PSS)	2 weeks	2 week PTSD severity

*PTSD* post-traumatic stress disorder, *ASD* acute stress disorder, *n* number of participants included at final assessment, *CAPS* clinician Administered PTSD scale, *IES-R* impact of events scale - revised, *DTS* Davidson trauma scale, *PCL-C* PTSD checklist - civilian version, *PCL - M* PTSD checklist - military version, *PSS* posttraumatic symptom scale, *MR* memory reactivation, *RTM* reconsolidation of traumatic memories.

^a^Included in meta-analysis.

**Table 2:** Characteristics of included consolidation studies.

Study	Country	Trauma sample	Consolidation procedure	Timing after trauma	Comparator	No. of sessions/dose	*n*	PTSD/ASD outcome	Follow up	Primary Outcome
Adult pharmacological consolidation										
Delahanty et al. 2013^a^	USA	Injury	Hydrocortisone	<12 h	Placebo	20 mg BD PO 10/7 plus 6/7 taper	43	PTSD severity (CAPS)	1, 3 months	1 & 3 month PTSD severity
Denke et al. 2008^a^	Germany	Septic Shock	Hydrocortisone	<6 h	Placebo	50 mg QDS IV 5/7 plus 6/7 taper	18	PTSD incidence (PTSS-10)	12 months	12 month PTSD incidence
Schelling et al. 2001^a^	Germany	Septic Shock	Hydrocortisone	<6 h	Placebo	100 mg IV bolus, plus 12/7 continuous infusion 12/7 and 6/7 taper	20	PTSD incidence & severity (SCID-IV & PTSS-10)	31 months	31 month PTSD incidence & severity
Schelling et al. 2004	Germany	Cardiac Surgery	Hydrocortisone	<6 h	Standard therapy	100 mg IV bolus, plus 2/7 continuous infusion and 2/7 taper	48	PTSD severity (PTSS-10)	6 months	6 month PTSD severity
Weis et al. 2006^a^	Germany	Cardiac Surgery	Hydrocortisone	<6 h	Placebo	100 mg IV bolus, plus 2/7 continuous infusion and 2/7 taper	28	PTSD incidence (PTSS-10)	6 months	Duration of intensive care unit treatment
Zohar et al. 2011^a^	Israel	Injury	Hydrocortisone	<6 h	Placebo	100–140 mg IV bolus	17	PTSD incidence (CAPS)	3 months	3 month PTSD incidence
Kok et al. 2016^a^	Netherlands	Cardiac Surgery	Dexamethasone	<6 h	Placebo	1 mg/kg IV bolus	2458	PTSD incidence (PTSS-10)	18–48 months	18–48 month PTSD incidence
Hoge et al. 2012^a^	USA	Injury	Propranolol	4–12 h	Placebo	120 mg BD PO 10/7 plus 9/7 taper	41	PTSD incidence & severity (CAPS)	1, 3 months	Physiological reactivity during script-driven traumatic imagery
Pitman et al. 2002^a^	USA	Injury	Propranolol	<6 h	Placebo	40 mg QDS PO 10/7	24	PTSD incidence & severity (CAPS)	1, 3 months	1 & 3 month PTSD incidence & severity
Stein et al. 2007^a^	USA	Injury	Propranolol	<48 h	Placebo	20 mg TDS PO 2/7, 40 mg TDS PO 8/7, plus 4/6 taper	38	ASD severity & PTSD incidence (ASDS & PCL-C)	1, 4, 8 months	1, 4 & 8 month ASD severity & PTSD incidence
Van Zuiden et al. 2017^a^	Netherlands	Injury	Oxytocin	6–12 h	Placebo	40IU BD intranasal 8/7	107	PTSD incidence & severity (CAPS)	1.5, 3, 6 months	1.5, 3 & 6 month PTSD incidence & severity
Child/Adolescent pharmacological consolidation										
Nugent 2007	USA	Injury	Propranolol	<12 h	Placebo	2.5 mg/kg per day	20	PTSD incidence & severity (CAPS-CA)	6 weeks	6 week PTSD incidence & severity
Adult psychological consolidation										
Freedman et al. 2020^a^	Israel	Injury	Virtual reality pain task	<8 h	No intervention	1	55	PTSD severity (PSS)	2 weeks, 6 months	2 weeks & 6 month PTSD severity
Horsch et al. 2017^a^	Switzerland	Caesaraen section	Cognitive task memory interference procedure conducted at the site of trauma	<6 h	No intervention	1	56	ASD severity, PTSD severity (ASDS & PDS) & intrusive memory frequency	1 week, 1 month	1 week intrusive memory frequency
Iyadurai et al. 2018^a^	UK	Injury	Cognitive task memory interference procedure with MR	<6 h	Attention-placebo control	1	71	PTSD incidence & severity (IES-R & PDS) & intrusive memory frequency	1 week, 1 month	1 week intrusive memory frequency
Kanstrup et al. 2021^a^	Sweden	Injury	Cognitive task memory interference procedure with MR	<72 h	Podcast control	1	41	PTSD incidence & severity (IES-R & MINI) & intrusive memory frequency	1 week, 1, 3, 6 months	1 week intrusive memory frequency

*PTSD* post-traumatic stress disorder, *ASD* acute stress disorder, *n* number of participants included at final assessment, *CAPS* clinician administered PTSD scale,

*PTSS-10* post-traumatic 10 stress symptom inventory, *IES-R* impact of events scale - revised, *SCID-IV* structured clinical interview for DSM IV, *ASDS* acute stress disorder scale,

*PCL-C* PTSD checklist - civilian version, *PSS* post-traumatic symptom scale, *PDS* post-traumatic diagnostic scale, *CAPS-CA* clinician-administered PTSD scale for children and adolescents, *MINI* mini-international neuropsychiatric interview, *mg* milligrams, *IU* international units, *BD* twice daily, *TDS* three times daily, *QDS* four times daily, *IV* intravenous, *PO* oral administration.

^a^Included in meta-analysis.

All studies assessed PTSD incidence (seven RCTs) or severity (18 RCTs) and of the prevention studies, only Stein et al. [43] and Horsch et al. [50] also assessed ASD, with three other studies [17, 51, 53] also assessing traumatic stress symptoms at 1-week post-trauma (symptoms must be present for 1 month following trauma to diagnose PTSD), the latter two doing so deliberately as a primary outcome since their target was this and not full PTSD. Seven studies also reported re-experiencing subscales for their PTSD outcomes [16, 17, 34, 48, 50–52] with three also reporting intrusive memories via a paper diary (not the syndrome of PTSD) as their primary outcome measures [50, 53, 54]. One study [14] reported that ‘IES-R intrusion and arousal scores did not differ between the treatment groups’ but did not provide subscale data.

Only eight studies also measured depressive symptom outcomes [10, 14, 17, 35, 43, 44, 46, 50], with four trials also assessing anxiety outcomes [17, 44, 46, 50] and two RCTs measuring health related quality of life (HRQOL) [36, 45]. Of these, only three hydrocortisone trials found differences in depression [35, 46], anxiety [35, 46] or HRQOL [35, 45].

When measuring outcomes from all trials, a clinician-administered measure was used where available (nine studies [10, 13, 35, 37, 39–41, 44, 46] and self-report questionnaires if not (16 studies).

### Risk of bias assessments

The quality of these RCTs was highly variable and the methodology of most studies had significant risk of bias (Supplementary Tables 2 and  3).

Only nine trials used intention-to-treat analyses [13, 16, 17, 34, 38, 44, 47, 50, 52],with the other 16 trials either using an unspecified or completer only analysis.

### Meta-analyses

**Fig. 2:**
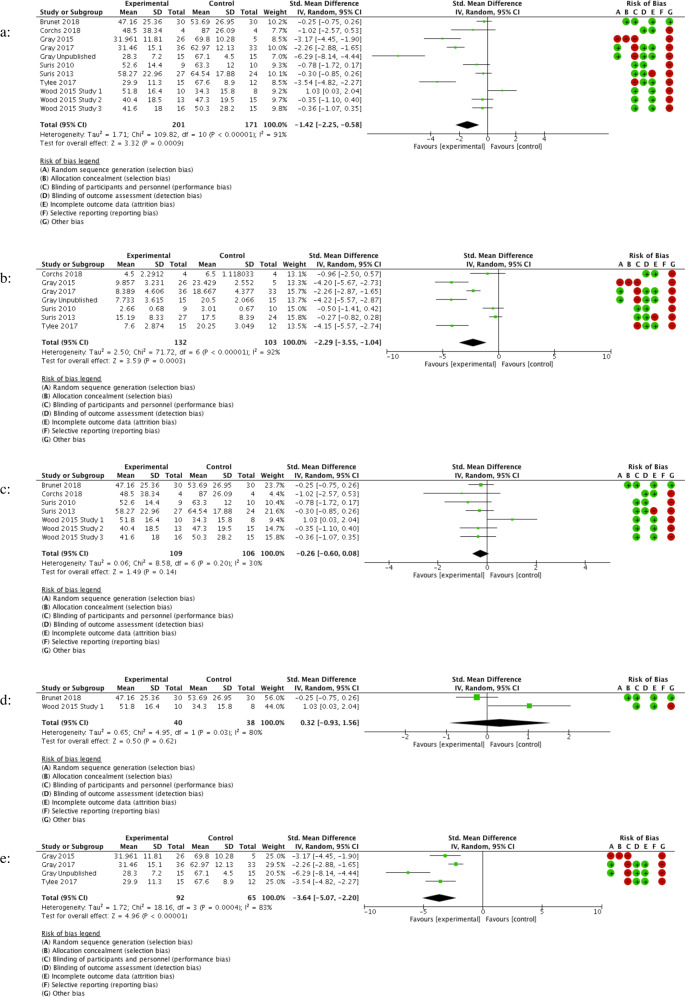
Forest plots of effects of reconsolidation therapies for PTSD treatment in adult participant RCTs. **a** All pharmacological/ECT/psychological reconsolidation interventions assessing PTSD severity at 1–4 weeks. **b** All pharmacological/ECT/psychological reconsolidation interventions assessing re-experiencing severity at 1–4 weeks. **c** All pharmacological/ECT reconsolidation interventions assessing PTSD severity at 1–4 weeks. **d** Propranolol and memory reactivation interventions assessing PTSD severity at 1 week. **e** RTM interventions assessing PTSD severity at 2 weeks.

**Fig. 3:**
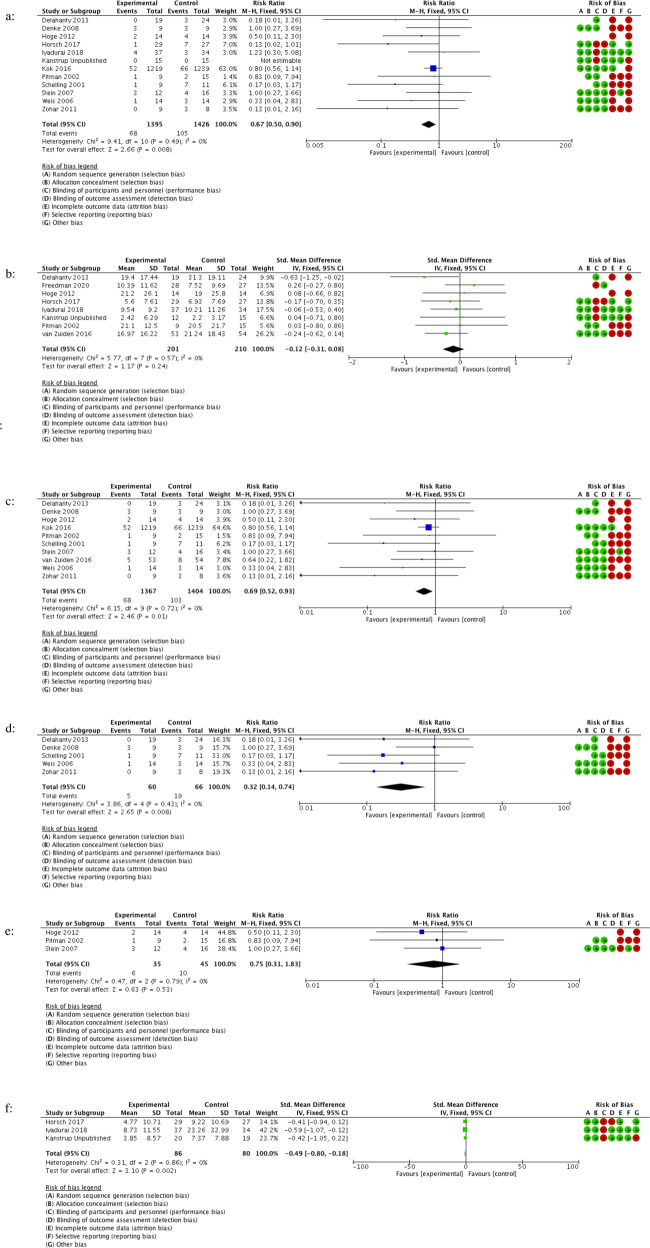
Forest plots of effects of consolidation therapies for PTSD/re-experiencing/intrusive memory prevention in adult participant RCTs. **a** All pharmacological/psychological consolidation interventions assessing PTSD incidence at 1–48 months. **b** All pharmacological/psychological consolidation interventions assessing PTSD severity at 2 weeks–6 months. **c** All pharmacological consolidation interventions assessing PTSD incidence at 3–48 months. **d** Hydrocortisone interventions assessing PTSD incidence at 3–31 months. **e** Propranolol interventions assessing PTSD incidence at 3–6 months. **f** Cognitive task memory interference procedure with MR interventions assessing intrusive memory severity at 1 week.

The results of our meta-analyses are shown in Tables 3 and 4, with associated forest plots in Figs. 2 and 3. A large effect was found for RTM versus waitlist placebo in four trials as well as for consolidation hydrocortisone versus placebo in five RCTs. CMTR was superior to control in preventing intrusive memories in three RCTs.

**Table 3:** Effects of reconsolidation therapies for PTSD treatment in adult participant RCTs.

Reconsolidation procedure	Outcome	Comparisons	Participants (*n*)	RR/SMD (95% CI)	I^2^	GRADE Judgement
All pharmacological/ECT/psychological reconsolidation interventions	PTSD severity 1–4 weeks	11	372	SMD: −1.42 (−2.25 to −0.58)	91%	Very low
All pharmacological/ECT/psychological reconsolidation interventions	Re-experiencing severity 1–4 weeks	7	235	SMD: −2.29 (−3.55 to −1.04)	92%	Very low
All pharmacological/ECT reconsolidation interventions	PTSD severity 1–4 weeks	7	215	SMD: −0.26 (−0.60, 0.08)	30%	Very low
propranolol & MR	PTSD severity 1 week	2	78	SMD: 0.32 (−0.93 to 1.56)	80%	Very low
RTM	PTSD severity 2 weeks	4	157	SMD: −3.64 (−5.07 to −2.20)	83%	Very low
RTM	Re-experiencing severity 2 Weeks	4	157	SMD: −3.60 (−4.85 to −2.35)	78%	Very low

*PTSD* post-traumatic stress disorder, *n* number of participants included at the final assessment, *RR* relative risk,

*SMD* standard mean difference, *CI* confidence interval, *ECT* electroconvulsive therapy, *MR* memory reactivation, *RTM* reconsolidation of traumatic memories.

**Table 4:** Effects of consolidation therapies for PTSD/Re-experiencing/intrusive memory prevention in adult and child/adolescent participant RCTs.

Consolidation procedure	Outcome	Comparisons	Participants (*n*)	RR/SMD (95% CI)	I^2^	GRADE judgement
Adult studies						
All pharmacological/psychological consolidation interventions	PTSD incidence 1–48 Months	12	2821	RR: 0.67 (0.50 to 0.90)	0%	Low
All pharmacological/psychological consolidation interventions (without hydrocortisone)	PTSD incidence 1–48 Months	7	2695	RR: 0.75 (0.55 to 1.03)	0%	Low
All pharmacological/psychological consolidation interventions	PTSD severity 2 weeks–6 months	8	411	SMD: −0.12 (−0.31, 0.08)	0%	Low
All pharmacological/psychological consolidation interventions	Re-experiencing severity 2 weeks–48 months	6	1421	SMD: −0.12 (−0.37, 0.13)	54%	Low
All pharmacological consolidation interventions	PTSD incidence 3–48 months	10	2771	RR: 0.69 (0.52 to 0.93)	0%	Low
All pharmacological consolidation interventions	PTSD severity 3–6 months	4	202	SMD: −0.25 (−0.53 to 0.03)	0%	Low
Hydrocortisone	PTSD incidence 3–31 months	5	126	RR: 0.32 (0.14 to 0.74)	0%	Low
Propranolol	PTSD incidence 3–6 months	3	80	RR: 0.75 (0.31 to 1.83)	0%	Low
Virtual reality pain task	PTSD severity 6 months	1	55	SMD: −0.46 (−0.99 to 0.08)	N/A	Very low
Virtual reality pain task	Re-experiencing severity 6 months	1	55	SMD: 0.14 (−0.39 to 0.67)	N/A	Very low
Cognitive task memory interference procedure with MR	PTSD severity 2 weeks–6 months	3	154	SMD: −0.08 (−0.40 to 0.23)	0%	Low
Cognitive task memory interference procedure with MR	PTSD incidence 1–6 Months	3	157	RR: 0.45 (0.05 to 4.18)	69%	Low
Cognitive task memory interference procedure with MR	Re-experiencing severity 4 weeks	2	127	SMD: −0.25 (−0.60 to 0.10)	0%	Low
Cognitive task memory interference procedure with MR	Intrusive memory frequency 1 week	3	166	SMD: −0.49 (−0.80 to −0.18)	0%	Low
Child & adolescent studies						
Propranolol^a^	PTSD severity 1–3 months	1	20	SMD: 0.01 (−0.87 to 0.89)	N/A	Very low

*PTSD* post-traumatic stress disorder, *n* number of participants included at final assessment, *RR* relative risk,

*SMD* standard mean difference, *CI* confidence interval, *MR* memory reactivation.

^a^Only one study for the outcome so data not pooled.

## Discussion

This meta-analysis assessed the efficacy of so-called, consolidation/reconsolidation therapy RCTs (although we must remain agnostic about the true underlying mechanism of action) in the prevention and treatment of PTSD, PTSD symptoms and intrusive symptoms. We found evidence of a large effect of psychological and pharmacological reconsolidation interventions, when grouped together, in the treatment of PTSD symptoms, and a smaller positive effect of psychological and pharmacological consolidation interventions in the prevention of PTSD. There was a wide range in the efficacy of the compared treatments. This is the first meta-analysis to assess the impact of consolidation and reconsolidation therapies on the prevention and treatment of PTSD (and symptoms of re-experiencing) and identified three interventions with promising emerging evidence (RTM), consolidation hydrocortisone and cognitive task memory interference procedure with MR).

Of reconsolidation RCTs, RTM was the only psychological reconsolidation intervention assessed here (with other protocols not meeting RCT inclusion criteria e.g. the rewind technique [15]). RTM demonstrated a large effect size in the treatment of PTSD symptoms. These trials, however, had high risk of bias, using nonrandom sampling in veteran populations with inadequately powered control groups and achieving a GRADE rating of very low. The external validity of these results in non-combat exposed populations remains unclear but the evidence suggests that evaluation in higher quality RCTs is warranted. Confirmation of efficacy is of particular clinical importance considering the already common use of the therapy [55] in addition to the possible advantages over TF-CBT (fewer sessions, non-disclosure of trauma but still trauma-focused). Excluding RTM trials in sensitivity analysis reduced the effect of reconsolidation interventions considerably (SMD: −0.26 (95% CI: −0.60 to 0.08)). RTM employs psychological techniques to decrease autonomic arousal whilst mobilising the traumatic memory (viewing the trauma in the third person, in black and white) before rapidly viewing the trauma in reverse and returning to a safe starting point. RTM’s greater involvement of cognitive manipulation (via rewinding techniques) and more detailed MR than the other interventions assessed here may account for some of these positive findings.

We found no overall effect of pharmacological/ECT reconsolidation agents plus MR (memory reminder) procedures on PTSD symptoms, nor any specific effect of propranolol plus MR in subgroup analysis. While Brunet et al. [13] compared propranolol and MR with placebo and MR (finding positive results in the intervention group), Wood et al. [49] compared propranolol and MR with propranolol without MR (finding no significant difference between groups). The dose and dose timing were identical in both studies, but Brunet et al. [13] used six propranolol/MR sessions, documenting gradual reductions in PTSD severity over the 6 weeks, while Wood et al. only used a singular session. The two studies [13, 49] also used different MR procedures. It is also possible that participants entering a clinical context for the treatment of their distressing memories are reminded of their trauma and this may mobilise traumatic memories even without formal MR. If this occurred in the Wood et al study, the control group would have been compromised. Evidently, uncertainty remains about the direct comparability of these two studies with neither study evaluating each element of the intervention in isolation (e.g. placebo/MR vs propranolol/MR vs propranolol without MR).

The negative results of certain reconsolidation studies highlight the specific limitations which exist when considering a therapeutic protocol’s ability to destabilise and modify a traumatic memory, and the importance of these boundary conditions [56]. The type of MR procedure used is likely to be crucial in determining the outcome. Optimal MR needs to be delineated—at present different groups interpret this differently e.g. may involve recalling the traumatic event in as much detail as the participant is able (without causing intolerable distress), including a mixture of visual images and bodily sensations e.g. touch, sound, smell. Or it may involve a milder stimulation—since there are no objective assays to measure if studies have insufficiently or excessively reactivated memories during reconsolidation [1] further behavioural research is needed to determine optimal parameters.

Our analysis of ten reconsolidation protocols identified four different types of MR: a more extensive written trauma narrative; a shorter written trauma native; audio-recorded trauma narratives; describing the trauma aloud). Future research should seek to administer MR procedures/reconsolidation agent timings with greater specification and to facilitate the likelihood of sufficient reactivation of traumatic memories to allow more confidence in either positive or negative findings of other reconsolidation agents. Suris et al. [10] found no significant difference between sirolimus/MR vs placebo/MR using a comparatively brief MR protocol, but also initiated MR prior to peak bloodstream sirolimus levels (peak sirolimus levels at 1-h post administration, with some participants receiving MR 30 minutes post). It remains unclear if these negative findings are a result of a failure to achieve MR, the blood concentration of sirolimus being too low to cause effective reconsolidation, or the failure of sirolimus to effectively reconsolidate traumatic memories even at peak blood level. Brunet et al. [13], overcame this issue by administering short and long-acting propranolol concurrently, with the timing of MR 15 min post peak systemic concentration of propranolol.

Our analyses demonstrate a weak positive effect of psychological/pharmacological consolidation interventions in preventing PTSD. Hydrocortisone was the only consolidation agent to demonstrate a positive effect on PTSD incidence, as confirmed by and discussed more extensively in previous reviews [22, 57], though not all studies analysed here were designed to examine PTSD incidence. Sensitivity analysis removing five hydrocortisone trials found no effect of other agents on PTSD incidence (SMD: 0.75 (95% CI: 0.55 to 1.03)). While the agents examined here appear suboptimal, future consolidation research could re-purpose promising reconsolidation therapies (e.g. delivering RTM within 6–12 h post-trauma).

Our meta-analysis documents the failure of propranolol to alter memory consolidation in the 12 h following trauma, further raising questions about the translation of consolidation theory into human participants. It may be that even during the consolidation phase MR may still be required in order to prevent PTSD with propranolol. The consolidation window could be as short as 6 h [46] although positive results of hydrocortisone administration between 6–12 hours suggest otherwise, or that consolidation is not responsible [35]. Our results clearly show that MR is not required to prevent PTSD with hydrocortisone if administered within 6–12 hs of the traumatic event and this is consistent with consolidation theory which suggests the memory is already labile in the immediate aftermath of trauma. The smaller consolidation prevention effect sizes demonstrated here compared to treatment are typical of prevention and screening studies more broadly, which require larger samples and greater power to detect smaller effects due to natural recovery [58]. The propranolol studies may have been underpowered to detect a small effect size (with our analyses only including 80 participants across three RCTs). The ability to deliver adequately powered consolidation studies is hampered by the practical limitations of identifying, consenting and enroling a participant into an RCT 6–12 h post-trauma. The small effect sizes achieved in pharmacological consolidation studies mirrors the results of pharmacotherapy treatment in PTSD [33].

Three of the four included behavioural consolidation studies demonstrated a small positive effect in reducing intrusive memories at 1-week post-trauma (the primary outcome) [17, 50, 53] via a mechanistically derived imagery competing task intervention. However, these studies were not powered to assess symptoms of PTSD at 1 month nor broader re-experiencing symptoms. Neither cognitive task memory interference procedure with MR, a virtual reality pain task protocol, nor RTM improved re-experiencing symptoms more than PTSD as a whole. It is noted that since studies were neither designed nor powered to examine these questions, this limits the interpretations that can be drawn. Therefore, it is currently not possible to assess amelioration of re-experiencing and other PTSD symptoms via improved intrusive memories, although future studies powered to do this are warranted.

While Iyadurai et al. [17] and Kanstrup et al. [53] investigated procedure including an MR plus cognitive task memory interference (i.e. mental imagery competing task), Horsch et al. [50] conducted the cognitive task memory interference at the site of trauma, providing an in vivo trauma reminder i.e. via an MR contextual cue (here the same hospital ward as the trauma occurred) (i.e. under the assumption there was no need for a separate verbal MR procedure). Horsch et al. [50] demonstrated a slightly greater treatment effect on PTSD symptoms and it may be possible that more detailed trauma reminders/MR (as per RTM, or the in vivo reminder of Horsch et al. [50]) may produce more promising results in other settings. In contrast to the MR procedure used by Brunet et al. [13], Suris et al. [14] and Corchs et al. [47], Iyadurai et al. [17] used a shorter procedure focused on intrusive image hotspots [59]. As the latter intervention was administered within the consolidation window, we cannot determine if this shorter memory reminder (rather than MR) can successfully orient to the specific memory [2], although further non randomised work suggests that possibility [53, 60–62].

Freedman et al. [51] used a virtual reality-based task utilising 'SnowWorld' rather than Tetris (unlike Horsch et al. [50], Iyaduarai et al. [17], Kanstrup et al. [53]) to produce a form of distraction originally developed for patients with burn injuries, e.g. cold and soothing. It is not clear if Snow World provides sufficient distraction to disrupt reconsolidation, nor whether it is visuospatial, and no mental rotation instructions (cognitively rotating objects to enhance visuospatial demands) were included (which would limit its efficacy as mental imagery competing task). Furthermore, Freedman et al. [51] did not include an MR procedure, nor do these components appear to have been administered within strict time parameters. Freedman et al. [51] used only 10 min of SnowWorld compared to 10–20 min of Tetris used in Horsch et al. [50] and Iyadurai et al. [17], which may again affect consolidation and thus the outcome. The less promising results of Freedman et al. [51] (compared to Horsch et al. [50] and Iyadurai et al. [17]) suggest that the details of the protocol (type of visuospatial task, MR procedure, timing parameters, and/or the presence of mental rotation) may be ingredients each of which can affect results. Future work should be informed by manualising protocols alongside training in procedures to capture the details of successful therapies e.g. utilising MR (or an in vivo trauma reminder), mental rotation, strict timing windows and use of Tetris (or other particularly engaging tasks) for visuospatial distraction. Tetris is especially absorbing, easily creating a sense of ‘flow’ which may provide sufficient diversion to allow memory updating [63], which SnowWorld may lack. The version of Tetris used (‘marathon mode’) progressively becomes harder, automatically personalising the intervention according to individual thresholds for optimal challenge and distraction and so may be particularly well suited to this application.

While consolidation and reconsolidation occur at different time frames in relation to the traumatic event, aspects of the underlying mechanism may share similarities. Thus, it may be possible that the positive findings from consolidation agents could be adapted to the reconsolidation phase with added MR. Suris et al. [14] attempted this with hydrocortisone, finding lower PTSD severity scores in the experimental group, albeit not reaching statistical significance in a small sample. These results suggest that hydrocortisone is the most promising pharmacological consolidation agent, with RTM the most promising reconsolidation therapy and with, by far, the largest effect size. Other reviews [22] have concluded that hydrocortisone could be considered as a preventative intervention for individuals with severe physical illness/injury within 6–12 h of a traumatic event, providing there are no contraindications to administration. Despite positive effects in heterogenous populations, future research should clarify the particular subgroups hydrocortisone is likely to be particularly efficacious in [38] and clarify the optimal dosing, dosing window and route [22].

Many of the pharmacological and psychological protocols we have examined in this study have broad neurobiological effects that target a variety of psychological processes, affecting PTSD outcomes through a multitude of different mechanisms. Oxytocin, for example, is also an anxiolytic as well as likely altering memory processing [44]. We have attempted to isolate the effect of memory consolidation/reconsolidation by only including studies where the consolidation agent was given within the 12 h following trauma for preventative studies, and where the protocol followed a strict reconsolidation procedure (trauma reactivation combined with a pharmacological agent, or for psychological studies, a reasonable rationale for considering reconsolidation as a mechanism of action).

Many of the studies included lacked specific detail in the documented protocol to determine if the principles of reconsolidation theory (as described in basic scientific research) have been adhered to e.g. the MR procedure. Following such principles is assumed to be fundamental in order to ameliorate fearful memories via reconsolidation [2]. This paper highlights the gulf in translation between basic scientific and clinical research, the careful steps that are needed, with some therapies likely employing some elements of reconsolidation theory but not being described as such (e.g. EMDR) and others using the terminology of reconsolidation without clarification that the underlying mechanism includes reconsolidation (e.g. RTM). Optimising reconsolidation therapies will likely require more specific translational work which strictly adheres to the fundamental underlying conditions of reconsolidation, as demonstrated in pilot and basic science work. This process should clarify the necessary therapeutic components and parameters of these multi-part, complex interventions to prevent future premature RCTs failing to demonstrate positive or negative findings that can be attributed to methodological issues.

The majority of the RCTs included in our meta-analyses were small (and often likely underpowered) with most having multiple areas of concerning the risk of bias. Notably, this applied to the four trials evaluating RTM and the five trials assessing hydrocortisone, limiting our confidence in the available evidence to determine the true effect sizes of these interventions. Furthermore, some trials (e.g. the RTM RCTs) only assessed outcomes at 2 weeks and so were unable to demonstrate the stability of treatment effects.

Some analyses compared psychological with pharmacological treatment, a comparison limited due to inadequate blinding of psychological interventions, but of important scientific interest considering the supposed common mode of action. Other key limitations of the field include the inability of any study to objectively confirm MR in participants, in addition to the broad mechanism of actions of the pharmacological agents used. Analyses of some reconsolidation protocols were impeded by the unavailability of re-experiencing data and we recommend the publication of subscale data in future RCTs, whilst acknowledging power issues associated with this. This meta-analysis does, however, compile a higher quality of PTSD/re-experiencing RCT-specific evidence than previous reviews [54, 64, 65] by excluding those studies which did not follow a strict reconsolidation protocol.

Ten studies investigated post-treatment psychiatric comorbidity [14, 17, 35, 36, 38, 43–46, 50]. Eight of these investigated depression, five examined anxiety and three assessed HRQOL. The only significant differences observed between the intervention and control group were in those studies investigating hydrocortisone, with intervention groups reporting significantly fewer depressive symptoms [35, 46], anxiety symptoms [46] and improved HRQOL [35, 45]. The amelioration of these secondary outcome measures in addition to reductions in PTSD incidence add support to hydrocortisone’s efficacy as a consolidation agent. Future consolidation/reconsolidation trials should report both depression and PTSD outcomes to further clarify this relationship.

This review highlights promising emerging evidence of consolidation and reconsolidation therapies in the prevention and treatment of PTSD (RTM, consolidation hydrocortisone) and the prevention of intrusive memories (cognitive task memory interference procedure with MR), despite low confidence in the published evidence. More rigorous RCTs are required prior to further treatment recommendations.

Despite the promise of increased neuroscientific approaches to mental disorders this has largely failed to deliver new treatments [66] but reconsolidation theory is one area where some progress could be possible. Our analyses demonstrate the disappointing translation of reconsolidation protocols from bench to bedside, possibly due to non-adherence to specific boundary conditions (we were, however, unable to systematically examine whether adherence to boundary conditions predicted efficacy of treatment [67], or whether different procedures were used to destabilise old vs recent memories [68]). Translational research should clarify the therapeutic components and parameters of these multi-part, complex interventions to increase the likelihood of positive findings and more confidently interpret negative findings of putative reconsolidation agents. Future research should continue to empirically evaluate the promising interventions of consolidation hydrocortisone, cognitive task memory interference procedure with MR and RTM and consider the development of novel approaches informed by existing research. Future research should continue to empirically evaluate the promising interventions of consolidation hydrocortisone, cognitive task memory interference procedure with MR and RTM. Sample sizes should be sufficiently powered. Investigation of other protocols should utilise optimal MR, administer MR during peak pharmacological concentration, utilise multiple sessions with appropriate control groups, report re-experiencing subscales and consider the repurposing of reconsolidation agents in the consolidation window (e.g. RTM 6–12 h post-trauma).

## Supplementary information

Search Strategy

Supplementary Tables 1-3
