# Supplementary material for: Consolidation/reconsolidation therapies for the prevention and treatment of PTSD and re-experiencing: a systematic review and meta-analysis
Source: Transl Psychiatry. 2021 Sep 3;11:453. doi: 10.1038/s41398-021-01570-w (PMC8417130; doi:10.1038/s41398-021-01570-w)
Supplement: Supplementary file 1 — Search Strategy [file 41398_2021_1570_MOESM1_ESM.docx]

Supplementary Information - Search strategy

For this review we used a search already undertaken for a number of other systematic reviews carried out to update the ISTSS Treatment Guidelines (ISTSS 2018). The original search strategy used PubMed, PsycINFO, Embase and the Cochrane database of randomised trials, with no limitation on start date.

Terms referring to PTSD were combined with terms referring to reconsolidation-based therapies (using both MeSH terms and text words), for example, the Rewind technique, Reconsolidation of Traumatic Memories (RTM) and cognitive task memory interference procedure with MR. We also checked the references of reviews of reconsolidation therapies (e.g Iyadurai et al. 2019, Walsh et al. 2018, Bolsoni & Zuardi 2019) and of recent systematic reviews and meta-analyses which have informed the development of guidelines for the prevention and treatment of PTSD, such as National Institute for Health and Care Excellence (NICE) and International Society for Traumatic Stress Studies (ISTSS) guidelines (NICE 2018, Bisson et al. 2019). Details of the searches and exact search strings are provided below.

Original search carried out on May 6, 2013 with updated search on March 20 2020.

PubMed:

MESH terms:

Stress Disorders, Traumatic [MeSH Terms] AND Prospective studies [MeSH Terms] AND Adrenergic beta- Antagonists [MeSH Terms] OR Dose-Response Relationship, Drug [MeSH Terms] OR Hydrocortisone/therapeutic use [MeSH Terms] OR Dexamethasone/therapeutic use [MeSH Terms] OR Oxytocin/therapeutic use [MeSH Terms] OR Memory Consolidation [MeSH Terms]

Stress Disorders, Traumatic [MeSH Terms] AND Adrenergic beta-Antagonists [MeSH Terms] OR Propranolol [MeSH Terms] OR Dose- Response Relationship, Drug [MeSH Terms] OR Hydrocortisone/therapeutic use [MeSH Terms] OR Dexamethasone/therapeutic use [MeSH Terms] OR Oxytocin/therapeutic use [MeSH Terms] OR Memory Consolidation [MeSH Terms]

PsycINFO

(TX Adrenergic beta-Antagonists OR TX beta-blocker OR TX hydrocortisone OR TX cortisol OR TX dexamethasone OR TX rewind OR TX RTM OR TX reconsolidation of traumatic memor* OR TX Tetris OR TX visuospatial intervention OR TX oxytocin OR TX reconsolidation OR TX consolidation) AND (post-traumatic stress disorders OR PTSD OR posttraumatic stress OR re-experiencing OR re-experiencing OR intrusions OR intrusive memor* OR involuntary memor*)

Embase

Keywords: ('trauma' OR 'anxiety' OR 'posttraumatic' OR 'post-traumatic' OR ‘PTSD’ OR ‘intrusions’ OR ‘intrusive memor*’ OR ‘re-experiencing’ OR ‘re-experiencing’ OR ‘involuntary memor*’) AND ('propranolol' OR ‘beta-blocker’ OR ‘beta blocker’ OR ‘hydrocortisone’ OR ‘cortisol’ OR ‘dexamethasone’ OR ‘Rewind’ OR ‘RTM’ OR ‘Reconsolidation of Traumatic Memor*’ OR ‘Tetris’ OR ‘Visuospatial Intervention’ OR ‘Oxytocin’ OR ‘reconsolidation’ OR ‘consolidation’ ) 'posttraumatic stress disorder'/exp OR 'psychotrauma'/exp

Limits: #7 AND ('clinical article'/de OR 'clinical trial'/de OR 'controlled clinical trial'/de OR 'controlled study'/de OR 'drug dose comparison'/de OR 'evidence based medicine'/de OR 'evidence based practice'/de OR 'human'/de OR 'major clinical study'/de OR 'open study'/de OR 'prospective study'/de OR 'randomized controlled trial'/de OR 'randomized controlled trial (topic)'/de OR 'retrospective study’/de)

Search CCDAN Cochrane Central Register of Controlled Trials:

Keywords: (propranolol OR beta-blocker OR hydrocortisone OR cortisol OR dexamethasone OR oxytocin OR Rewind OR RTM OR Reconsolidation of Traumatic Memor* OR tetris OR visuospatial intervention OR reconsolidation OR consolidation) and (ptsd or (trauma AND stress) OR 'posttraumatic' OR 'post-traumatic' OR ‘PTSD' OR ‘intrusions’ OR ‘re-experiencing’ OR ‘re-experiencing’ OR ‘intrusive memor*’ OR ‘involuntary memor*’ ) (Limit; trials)

Additional searches:

Reference lists of earlier narrative reviews of reconsolidation therapies in PTSD (e.g. Iyadurai et al. 2019, Walsh et al. 2018, Bolsoni & Zuardi 2019) and of recent systematic reviews and meta-analyses which have informed the development of guidelines for the prevention and treatment of PTSD (e.g. NICE, ISTSS guidelines).
