# Supplementary material for: Consolidation/reconsolidation therapies for the prevention and treatment of PTSD and re-experiencing: a systematic review and meta-analysis
Source: Transl Psychiatry. 2021 Sep 3;11:453. doi: 10.1038/s41398-021-01570-w (PMC8417130; doi:10.1038/s41398-021-01570-w)
Supplement: Supplementary file 2 — Supplementary Tables 1-3 [file 41398_2021_1570_MOESM2_ESM.docx]

| **Supplementary Table 1: PRISMA 2009 Checklist** | | | |
| --- | --- | --- | --- |
| **Section/topic** | **#** | **Checklist item** | **Reported on page #** |
| **TITLE** | | |  |
| Title | 1 | Identify the report as a systematic review, meta-analysis, or both. | 1 |
| **ABSTRACT** | | |  |
| Structured summary | 2 | Provide a structured summary including, as applicable: background; objectives; data sources; study eligibility criteria, participants, and interventions; study appraisal and synthesis methods; results; limitations; conclusions and implications of key findings; systematic review registration number. | 1 |
| **INTRODUCTION** | | |  |
| Rationale | 3 | Describe the rationale for the review in the context of what is already known. | 2 |
| Objectives | 4 | Provide an explicit statement of questions being addressed with reference to participants, interventions, comparisons, outcomes, and study design (PICOS). | 2 |
| **METHODS** | | |  |
| Protocol and registration | 5 | Indicate if a review protocol exists, if and where it can be accessed (e.g., Web address), and, if available, provide registration information including registration number. | 4 |
| Eligibility criteria | 6 | Specify study characteristics (e.g., PICOS, length of follow-up) and report characteristics (e.g., years considered, language, publication status) used as criteria for eligibility, giving rationale. | 5 |
| Information sources | 7 | Describe all information sources (e.g., databases with dates of coverage, contact with study authors to identify additional studies) in the search and date last searched. | 26 |
| Search | 8 | Present full electronic search strategy for at least one database, including any limits used, such that it could be repeated. | 26 |
| Study selection | 9 | State the process for selecting studies (i.e., screening, eligibility, included in systematic review, and, if applicable, included in the meta-analysis). | 4 |
| Data collection process | 10 | Describe method of data extraction from reports (e.g., piloted forms, independently, in duplicate) and any processes for obtaining and confirming data from investigators. | 5 |
| Data items | 11 | List and define all variables for which data were sought (e.g., PICOS, funding sources) and any assumptions and simplifications made. | 5 |
| Risk of bias in individual studies | 12 | Describe methods used for assessing risk of bias of individual studies (including specification of whether this was done at the study or outcome level), and how this information is to be used in any data synthesis. | 5 |
| Summary measures | 13 | State the principal summary measures (e.g., risk ratio, difference in means). | 5 |
| Synthesis of results | 14 | Describe the methods of handling data and combining results of studies, if done, including measures of consistency (e.g., I^2^) for each meta-analysis. | 5 |

| **Supplementary Table 2: Risk of bias assessments for reconsolidation studies** | | | | | | | | |
| --- | --- | --- | --- | --- | --- | --- | --- | --- |
|  | **Risk of bias criteria** | | | | | | | |
|  | **Random sequence generation** | **Allocation concealment** | **Blinding of participants and personnel** | **Blinding of outcome assessment** | **Incomplete outcome data** | **Selective reporting** | **Other sources of bias** | **Total no of low risk domains** |
| **Adult Pharmacological/ECT Reconsolidation** | | | | | | | | |
| Brunet et al. 2018 | Unclear | Low Risk | Low Risk | Unclear | Low Risk | Unclear | Low Risk | 4 |
| Wood et al. 2015 Study 1-3 | Unclear | Unclear | Low Risk | Unclear | Low Risk | Unclear | High Risk | 2 |
| Suris et al. 2010 | Unclear | Unclear | Low Risk | Low Risk | Unclear | Unclear | High Risk | 2 |
| Suris et al. 2013 | Unclear | Unclear | Low Risk | Low Risk | High Risk | Unclear | High Risk | 2 |
| Corchs et al. 2018 | Unclear | Unclear | Unclear | Low Risk | Low Risk | Unclear | High Risk | 2 |
| **Adult Psychological Reconsolidation** | | | | | | | | |
| Gray et al. 2017 | Unclear | Unclear | High Risk | Low Risk | Unclear | Unclear | Unclear | 1 |
| Gray et al. 2015 | High Risk | High Risk | High Risk | Unclear | Unclear | Unclear | High Risk | 0 |
| Gray et al. Unpublished | Low Risk | Unclear | High Risk | Low Risk | Low Risk | Unclear | High Risk | 3 |
| Tylee et al. 2017 | Unclear | Unclear | High Risk | Low Risk | Low Risk | Unclear | High Risk | 2 |

| **Supplementary Table 3: Risk of bias assessments for consolidation studies** | | | | | | | | |
| --- | --- | --- | --- | --- | --- | --- | --- | --- |
|  | **Risk of bias criteria** | | | | | | | |
|  | **Random sequence generation** | **Allocation concealment** | **Blinding of participants and personnel** | **Blinding of outcome assessment** | **Incomplete outcome data** | **Selective reporting** | **Other sources of bias** | **Total no of low risk domains** |
| **Adult Pharmacological Consolidation** | | | | | | | | |
| Delahanty et al. 2013 | Unclear | Unclear | Low Risk | Unclear | High Risk | Unclear | High Risk | 1 |
| Denke et al. 2008 | Low Risk | Low Risk | Low Risk | Unclear | High Risk | Unclear | High Risk | 3 |
| Schelling et al. 2001 | Unclear | Low Risk | Low Risk | Low Risk | High Risk | Unclear | High Risk | 3 |
| Schelling et al. 2004 | Unclear | High Risk | Unclear | High Risk | High Risk | Unclear | High Risk | 0 |
| Weis et al. 2006 | Low Risk | Low Risk | Low Risk | Low Risk | High Risk | Unclear | High Risk | 4 |
| Zohar et al. 2011 | Low Risk | Low Risk | Low Risk | Low Risk | High Risk | High Risk | High Risk | 4 |
| Kok et al. 2016 | Low Risk | Low Risk | Low Risk | Low Risk | Low Risk | Unclear | High Risk | 5 |
| Hoge et al 2012 | Unclear | Unclear | Unclear | Unclear | High Risk | Unclear | High Risk | 0 |
| Pitman et al. 2002 | Unclear | Low Risk | Low Risk | Unclear | High Risk | High Risk | High Risk | 2 |
| Stein et al 2007 | Low Risk | Low Risk | Low Risk | Low Risk | High Risk | Low Risk | High Risk | 5 |
| van Zuiden et al. 2017 | Low Risk | Low Risk | Low Risk | Low Risk | Low Risk | High Risk | High Risk | 4 |
| **Child/Adolescent Pharmacological Consolidation** | | | | | | | | |
| Nugent 2007 | Unclear | Low Risk | Low Risk | Low Risk | High Risk | Unclear | High Risk | 3 |
| **Adult Psychological Consolidation** | | | | | | | | |
| Freedman et al. 2020 | Unclear | Unclear | High Risk | Low Risk | Unclear | Unclear | Unclear | 1 |
| Horsch et al. 2017 | Low Risk | Low Risk | High Risk | High Risk | Low Risk | Unclear | Low Risk | 4 |
| Iyadurai et al. 2018 | Low Risk | Low Risk | High Risk | Low Risk | Low Risk | Low Risk | Low Risk | 6 |
| Kanstrup et al. Unpublished | Low Risk | Low Risk | High Risk | Low Risk | Low Risk | Low Risk | High Risk | 5 |
